# Endometrial Scratch (Injury): Does Timing Matter? 

**Published:** 2019-06

**Authors:** Abigail Bernard, Katelyn Schumacher, Courtney Marsh

**Affiliations:** 1 School of Medicine, University of Kansas, Kansas City, Kansas, USA; 2 Department of Obstetrics and Gynecology, School of Medicine, University of Kansas, Kansas City, Kansas, USA

**Keywords:** IVF, Endometrial Scratch, Endometrial Injury, Assisted Reproductive Technology

## Abstract

**Objective:** To examine the timing of endometrial scratch in a patient’s menstrual cycle and whether there is an association with subsequent implantation.

**Materials and methods:** This study is a retrospective chart review on women, aged 18-45, seen in a reproductive endocrine clinic seeking conception. Timing of endometrial scratch was defined as proliferative (cycle day 1-9), periovulatory (CD11-16), or secretory (CD19+). All periovulatory biopsies were performed at time of oocyte retrieval in women freezing all oocytes/embryos for future use. Primary outcome of interest was positive beta-hCG within ninety days of the endometrial scratch.

**Results:** Sixty-nine cases of endometrial scratch met the inclusion criteria. There were no statistically significant differences in baseline demographic characteristics between those who received endometrial injury in the three phases. There was no significant difference in frequency of positive beta-hCG within 90 days of endometrial scratch between the patients who received an endometrial scratch in the three phases (proliferative 65.6%, periovulatory 69.6%, secretory 64.3%; p = 0.9332).

**Conclusion:** In contrast to prior studies which showed up to 65% decrease in implantation rate after endometrial scratch performed at time of oocyte retrieval, this study shows no significant difference in implantation when the injury is performed at the time of oocyte retrieval as compared to other phases of the menstrual cycle. Possible explanation may be that we did not perform a scratch if fresh embryo transfer was planned. As endometrial injury is associated with patient discomfort, performing the scratch while under conscious sedation for oocyte retrieval may be desirable in cycles where fresh embryo transfer is not planned. Future studies are needed to assess the validity of these findings.

## Introduction

Implantation of the blastocyst is a complex, selective process that involves the interaction of a variety of molecules. The process of apposition and adhesion of the blastocyst and later trophoblast invasion depends upon a receptive endometrium. It is well known that this process is under the influence of steroid hormones (estrogen and progesterone), but the molecular basis of implantation is an active interest in the field. Successful implantation is vital and considered the rate-limiting step in in vitro fertilization (IVF) cycles that result in pregnancy. 

Various techniques have been suggested to improve endometrial receptivity and facilitate implantation, one being endometrial scratching (injury). There is no consensus in the literature of whether endometrial injury increases the probability of pregnancy in women undergoing IVF cycles. One study demonstrated that performing repeat endometrial injuries prior to the IVF treatment and embryo transfer yielded an increased rate of live births 99 (1). In a large randomized controlled trial, results suggested that endometrial scratching did not result in a higher rate of live birth than no intervention among women undergoing IVF (2).

Precise timing of the endometrial scratch has similarly yielded varying results in the literature. In previous randomized control trials, the timing of the endometrial injury varied, with some studies performing the injury between the cycle days 4-7 (3), between cycle days 16-23 (4), between cycle days 21-26 (5), or even performing the intervention twice within the same cycle (6). Furthermore, most studies examining endometrial injury perform the scratch in the luteal phase, which does not allow for women to attempt conception in the prior cycle. In spite of increased research output over the past fifteen years, there are persistent unknowns regarding the intervention.

Endometrial injury is associated with patient discomfort and may necessitate a separate visit outside of typical monitoring. In this study, we examine the timing of endometrial scratch and whether there is an association with subsequent implantation.

## Materials and methods

This study was a retrospective chart review that evaluated patient charts between February 2017 and April 2018 at the University of Kansas Center for Advanced Reproductive Medicine in Kansas City, Kansas. The patients were women, aged 18-45, who were seen in the reproductive endocrine clinic seeking conception. The inclusion criteria include: (i) subfertile women indicated for IVF who received an endometrial injury (per clinical recommendations from provider) in the aforementioned time frame and (ii) follow-up for at least 90 days after the endometrial injury. Women were excluded if they were lost to follow-up or had pathology on endometrial biopsy suggesting malignancy or hyperplasia. Consent was waived because patient care was not impacted and the study evaluated existing, already collected data. Data was entered directly into REDCap, a password-protected secure database located behind the University of Kansas Medical Center firewall. Institutional Review Board (IRB) approval was obtained. This research did not receive any specific grant from funding agencies in the public, commercial, or not-for-profit sectors. 

Analysis was done with the assistance of the Department of Preventive Medicine and Public Health at the University of Kansas Medical Center. ANOVA tests for continuous variables and Pearson’s chi square tests for categorical variables were used. If frequencies were small, then the Fischer exact test was used. p < 0.05 was used for determining statistical significance (Table 2). Sample size calculation was performed using difference in positive beta-hCG of 40% between groups, alpha 0.05, and power of 80%. 

Mechanical endometrial injury was performed by a physician or a nurse practitioner using an endometrial pipelle. The catheter was then introduced through the cervix into the uterine cavity. The piston of the catheter was withdrawn to create negative pressure and subsequent suction at the catheter tip. The operator twisted the catheter while moving the catheter further into the uterine cavity and then slightly withdrawing. The catheter was then removed and the endometrial injury was complete. 

The timing of the injury varied amongst subjects. The menstrual cycle that the women was in at the time of her scratch was estimated by determining the number of days between cycle day one (the first day of her last menstrual period) and the endometrial injury. Often, the patient’s cycle day one (CD1) was documented in the chart. If the CD1 was not documented, the start of ovarian stimulation medications was assumed to be CD3, and CD1 was determined from that date. The menstrual stages were defined as follows: proliferative as CD1-9, periovulatory as CD11-16, secretory as CD19 and beyond. All periovulatory biopsies were performed at time of the oocyte retrieval in women freezing all oocytes/embryos for future use. Secretory phase biopsies were performed in a cycle with barrier method contraception recommended (if indicated). 

The primary outcome of interest was a positive beta-hCG blood test within 90 days of the endometrial scratch. Secondary outcomes were miscarriage, ectopic, and ongoing clinical pregnancy. Ongoing clinical pregnancy was defined as presence of cardiac activity on ultrasound.

## Results

Sixty-nine cases of endometrial scratch with subsequent embryo transfer met the inclusion criteria and were analyzed. 46.4% (n = 32) of the injuries occurred in the proliferative phase, 33.3% (n = 23) of the injuries were in the periovulatory phase, and 20.3% (n = 14) occurred in the secretory phase. The 69 endometrial injuries occurred in 62 patients. There were 7 instances in which a patient received 2 injuries in the allotted time frame. Each instance was treated as an independent event.

**Table 1:** Unique Patient Characteristics (n=62)

**Unique Patient Characteristic**	**Average Value**
Age	34.0 years
Body Mass Index (BMI)	26.9 kg/m²
	**Frequency, Percentage ** **of Unique Patients**
Multiparous	N = 32, 51.6%
At Least One Prior Miscarriage	N = 15, 24.2%

Unique patient characteristics (n = 62) are displayed in Table 1. The most common diagnosis and indication for IVF was male factor which occurred in greater than one-third of the unique patients included in this study (33.9%, n = 21). Of note, many patients had multiple diagnoses and indications. Additional prevalent diagnoses included: diminished ovarian reserve (32.3% n = 20), ovarian dysfunction (17.7%, n = 11), unexplained infertility (14.5%, n = 9) hypothyroidism (12.9%, n = 8), endometriosis (11.3%, n = 7), recurrent pregnancy loss (9.7%, n = 6), and tubal disease (9.7%, n = 6).

Between February 2017 and April 2018, 19.4% (n = 12) of the unique patients had undergone at least one prior embryo transfer at the University of Kansas Center for Advanced Reproductive Medicine prior to the cycle analyzed in this study. Four of these previous transfers resulted in a biochemical pregnancy, the others failed implantation. 

Between February 2017 and April 2018, 24.2% (n = 15) of the patients tried intrauterine insemination at the University of Kansas Center for Advanced Reproductive Medicine prior to the analyzed cycle. 12.9% (n = 8) of the patients tried ovulation induction with oral medications.

Table 2 shows case characteristics by phase of menstrual cycle in which the endometrial scratch was performed. The three groups were not significantly different in the age, BMI, or prevalence of Society for Assisted Reproductive Technology diagnoses.

As shown in Table 3, there was no significant difference in implantation rates between the patients who received an endometrial scratch in the proliferative, periovulatory, or secretory phase (p = 0.9332). Out of the 69 cases who received endometrial injury and subsequent embryo transfer, 46 cases (66.7%) had a positive serum beta-hCG within 90 days of the injury. Thirty-two (46.4%) of the 69 total cases had embryo transfers that resulted in an ongoing clinical pregnancy, 23 (33.3%) resulted in failed implantations, 9 (13.0%) resulted in biochemical pregnancies, and 5 (7.2%) resulted in spontaneous abortions. 

Of these 46 cases with a positive beta-hCG within 90 days of endometrial scratch, the majority resulted in an ongoing clinical pregnancy (69.6%, n = 32). The remaining cases resulted in biochemical pregnancies (19.6%, n = 9) or spontaneous abortions (10.9%, n = 5) (Table 3).

**Table 2:** Case Characteristics vs. Phase of Menstrual Cycle Receiving Scratch

**Case Characteristic**	**Proliferative** **n (row %)**	**Periovulatory** **n (row %)**	**Secretory** **n (row %)**	**P-value**	**Total** **n**
Age				p = 0.9130	
< 35 years	18 (48.7)	12 (32.4)	7 (18.9)		37
≥ 35 years	14 (43.75)	11 (34.4)	7 (21.9)		32
BMI				p = 0.9111	
≤ 25	13 (40.6)	11 (34.4)	8 (25.0)		32
> 25	13 (44.8)	10 (34.5)	6 (20.7)		29
SARTª Diagnosis					
Male factor	11 (47.8)	8 (34.8)	4 (17.4)	p = 0.9543	23
Diminished Ovarian Reserve	13 (61.9)	6 (28.6)	2 (9.5)	p = 0.1782	21
Ovarian Dysfunction	5 (38.5)	4 (30.8)	4 (30.8)	Pr <= p =.5208	13
Endometriosis	6 (66.7)	1 (11.1)	2.(22.2)	Pr <= P = 0.3659	**9**
Hypothyroidism	5 (55.6)	3 (33.3)	1 (11.1)	Pr <= p = 0.8977	9
Recurrent Pregnancy Loss	4 (50.0)	3 (37.5)	1 (12.50)	Pr <= p = 1.0	8
Unexplained Infertility	6 (54.6)	4 (36.4)	1 (9.1)	Pr <= p = 0.7626	11
Tubal Disease	5 (71.43)	2 (28.6)	0	Pr <= P = 0.3372	7

*Society for Assisted Reproductive Technology

**Table 3:** Phase of Cycle vs. Positive Beta-hCG within 90 Days of Endometrial Scratch

	**Beta-hCG + within 90 days of ** **Endometrial Scratch**		
	**Yes**	**No**	**Total**
Proliferative (CD1-9)	21 (65.6%)	11 (34.4%)	32 (46.4%)
Periovulatory (CD11-17)	16 (69.6%)	7 (30.4%)	23 (33.3%)
Secretory (CD19+)	9 (64.3%)	5 (35.7%)	14 (20.3%)
Total	46	23	69 p = 0.9332

Top Value: Frequency; Bottom Value: Row Percentage

There were no ectopic pregnancies. 12.9% (n = 8) of the unique patients had at least one previous endometrial scratch.

## Conclusion

In the current study, we evaluated whether the timing of the endometrial scratch was associated with differing subsequent implantation rates. Our results show that the menstrual phase in which the endometrial injury is performed is not associated with significantly different implantation rates. One previous study shows that implantation rates were significantly lower when endometrial injuries were performed at the time of oocyte retrieval (7). However, that study performed the injury in women undergoing a fresh embryo transfer where the embryo was transferred on day two after retrieval. In our study, periovulatory scratches performed at the time of oocyte retrieval were only performed in women who were forgoing a fresh transfer. Implantation rates in these cases were not significantly lower using this method. 

There are limitations of this study. Almost one-third of the unique patient population had diminished ovarian reserve, and therefore our results may not be generalizable. Additionally, there is a limitation in the nature of the study, being retrospective rather than a randomized control trial. 

The endometrial injury involves a pelvic exam, clinic visit, and a mild amount of pain. Based on our results there is no difference in subsequent implantation if the injury is performed in a freeze all cycle during the oocyte retrieval. There are benefits to performing the injury at this time. The patient is already accessing the clinic for the procedure, so they do not need an extra clinic visit, and they will not experience discomfort because they are anesthetized. Additionally, as the number of patients desiring assisted reproductive technology continues to increase, decreasing the need for additional clinic visits would be advantageous.
